# Necrosis and Neurotoxicity of the Brainstem Following Stereotactic Radiosurgery for Trigeminal Neuralgia: How Much is Too Much?

**DOI:** 10.1016/j.adro.2026.102103

**Published:** 2026-09-11

**Authors:** Timothy Chen, Michael Chaga, Wenzheng Feng, Tingyu Wang, Patrick Pema, Akil Anthony, Darra Conti, Jing Feng, Ma Rhudelyn Rodrigo, Elizabeth Luick, Joseph Hanley, Shabbar Danish

**Affiliations:** a Department of Radiation Oncology, Jersey Shore University Medical Center, Neptune, New Jersey; b Department of Neurosurgery, Hackensack Meridian School of Medicine, Hackensack, New Jersey; c Department of Genetics and Statistics, Rutgers University, New Brunswick, New Jersey; d Department of Neurosurgery, Jersey Shore University Medical Center, Neptune, New Jersey

## Introduction

Trigeminal neuralgia (TN) is a neurologic syndrome that presents with spontaneous episodes of severe electric shock-like pain along the trigeminal nerve dermatomes. Primary treatment strategies include antiseizure medication, surgical intervention such as microvascular decompression, and stereotactic radiosurgery (SRS). Given its effectiveness and noninvasive nature, SRS is advantageous for patients with surgical comorbidities or advanced age. Additionally, radiosurgery is associated with lower rates of retreatment and is widely available, which has driven the clinical adoption of TN SRS. The goal of TN SRS is to downregulate nociceptive ephaptic nerve transmission with precision ionizing radiation of the trigeminal nerve segment. Very high radiation dose is delivered to a small target near highly critical anatomy such as the brainstem, requiring maximal targeting accuracy and sharp dose falloff.^1^, ^2^

Radiosurgery for TN in patients with multiple sclerosis (MS) is an accepted therapy,^3^ although there has been evidence that patients with demyelinating diseases who receive SRS are at an increased risk of treatment-related neurotoxicity.^4^, ^5^ Here we report a MS patient treated with ZAP-X (ZAP Surgical Systems, Inc) TN SRS, who developed radiation necrosis in the brainstem and neurologic deficits following treatment, potentially due to an exaggerated inflammatory response triggered by SRS. The study protocol was reviewed and approved by the institutional review board, Pro2024-0029.

## Clinical Course

A 57-year-old female MS patient presented with bilateral facial pain TN since 2017, involving the left ventricle (V1) and V2 innervated areas and right V2 innervated area, with greater left side pain. The patient had previously been treated with gabapentin 400 mg 3 times daily, Tegretol 400 mg 3 times daily, baclofen 10 mg as needed, and peripheral nerve block. Current medication included gabapentin 400 mg 3 times daily and baclofen 10 mg as needed. Because the pain was refractory and magnetic resonance imaging (MRI) demonstrated no obvious compression lesion, the patient was not a candidate for microvascular decompression.^6^ The patient was recommended treatment with the ZAP-X platform. A clinical examination was performed at consultation by a qualified neurosurgeon and radiation oncologist consisting of direct evaluation and Modified Barrow Neurological Institute (BNI) pain scale classification.^7^ The patient BNI was classified as IV. Evaluation of the type of TN was made according to the classifications proposed by Eller et al^8^ and comprised idiopathic Type I and Type II. The patient was classified as Type I, described as sharp, shooting, electric shock-like pain that is present for more than 50% of the time with pain-free intervals.

**Figure 1:**
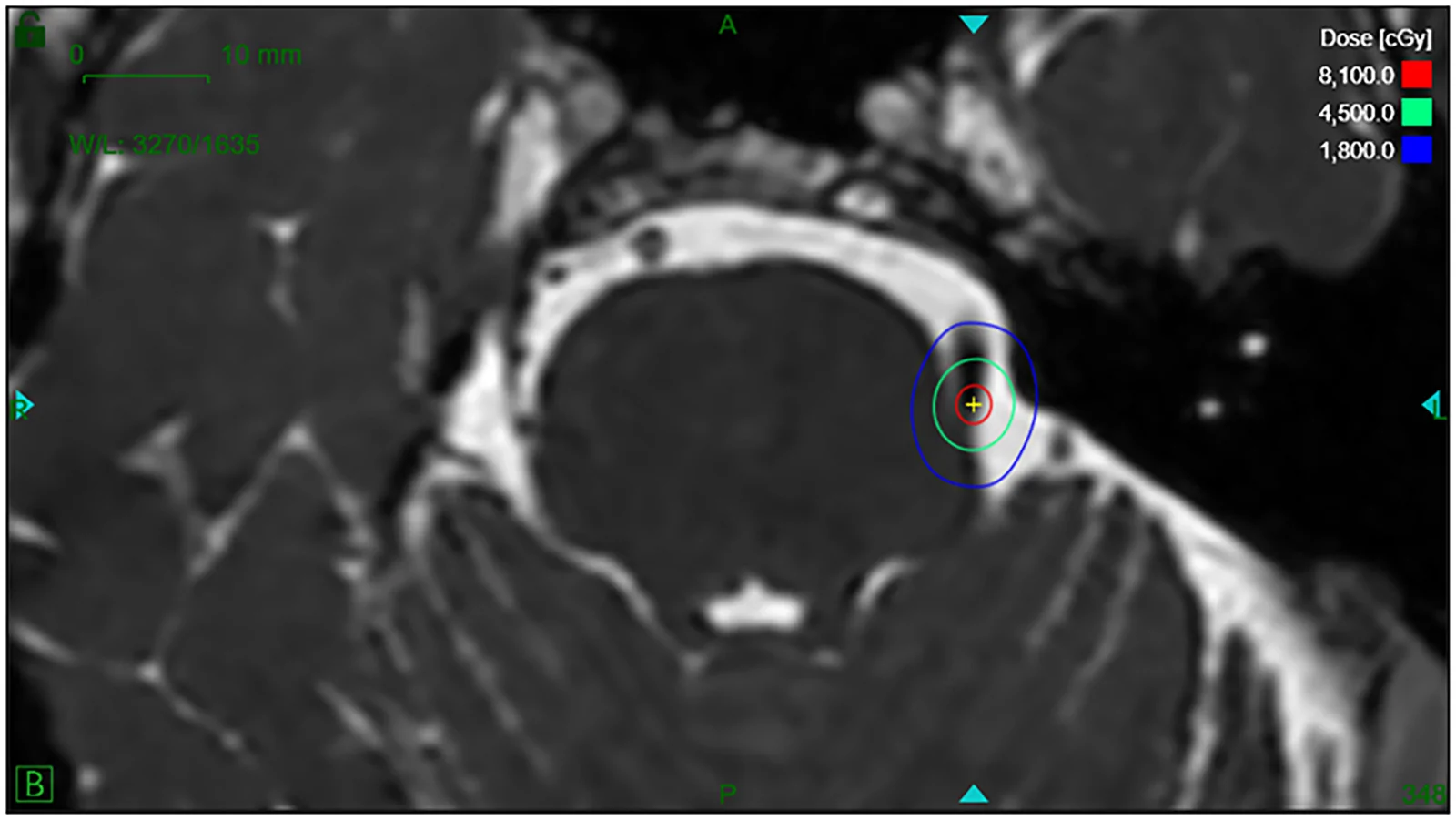
ZAP-X trigeminal neuralgia stereotactic radiosurgery with a target covering 0.03 cm^3^ of the trigeminal nerve in the entry zone location. A single isocenter was placed in the target with a prescription maximum dose of 9000 cGy to the 100% isodose line such that the 50% isodose line (green) contacted the brainstem. Isodose lines: red = 90%, green = 50%, blue = 20%.

For treatment planning, 9000 cGy was prescribed to 100% isodose line at the left dorsal root entry zone location. A single isocenter of 5-mm diameter was placed to have 50% isodose line (4500 cGy) abutting the brainstem after dose calculation (Fig. 1). The single-fraction treatment plan used path complexity of 6 for gantry movement. Forward planning was performed using a 0.5-mm dose grid, limiting the dose to the eyes, lens, optic nerves, optic chiasm, cochleae, brainstem, and spinal cord based on Timmerman organ-at-risk recommendations.^9^ The maximum point dose (D_max_) to the brainstem was 7874.77 cGy, the dose to 0.035 cm^3^ of the brainstem (D0.035cc) was 4140.4 cGy, and the brainstem volume receiving 1000 cGy was 0.446 cm^3^. The D0.035cc < 1500 cGy Timmerman brainstem constraint for 1 fraction was not met due to the root entry zone target location and the high dose delivered adjacent to the brainstem. The number of beams was 147. All plans were prepared and evaluated by a qualified radiation oncologist, neurosurgeon, and medical physicist. To ensure comfort and positioning, the patient was placed in thermoplastic mold care and an Efficast head and shoulder mask (Orfit Industries), using 2 shims.^10^, ^11^

Clinical follow-up visits were performed in-person by a qualified neurosurgeon and radiation oncologist approximately 1, 4, 7, and 8 months after treatment which consisted of direct evaluation, BNI pain classification, and follow-up MRI. Positive outcomes were defined as less than presentation BNI scale. Recurrence was defined as greater than last follow-up BNI scale.

**Figure 2:**
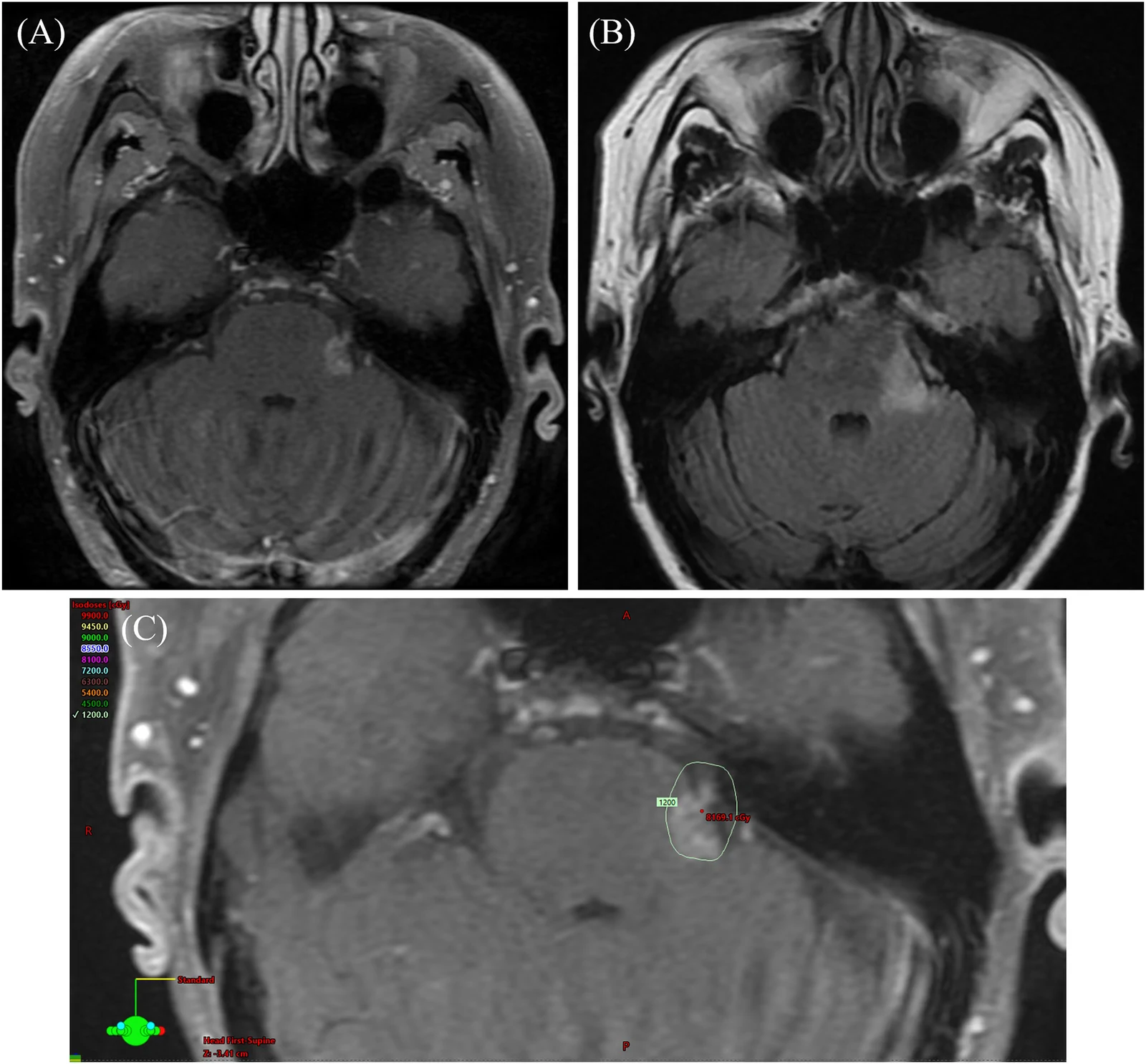
(A) Follow-up T1 magnetic resonance images with contrast yielded increased signal intensity in the lateral pons on the left side of the root entry zone. (B) Fluid-Attenuated Inversion Recovery (FLAIR) magnetic resonance imaging demonstrating greater area of enhancement and greater area of surrounding FLAIR hyperintensity at the pons root entry zone of the trigeminal nerve as well as a focal enhancement of the very proximal root of the left trigeminal nerve. (C) The T1-post and T2-FLAIR signal matched with the 1200 cGy isodose line.

**Figure 3:**
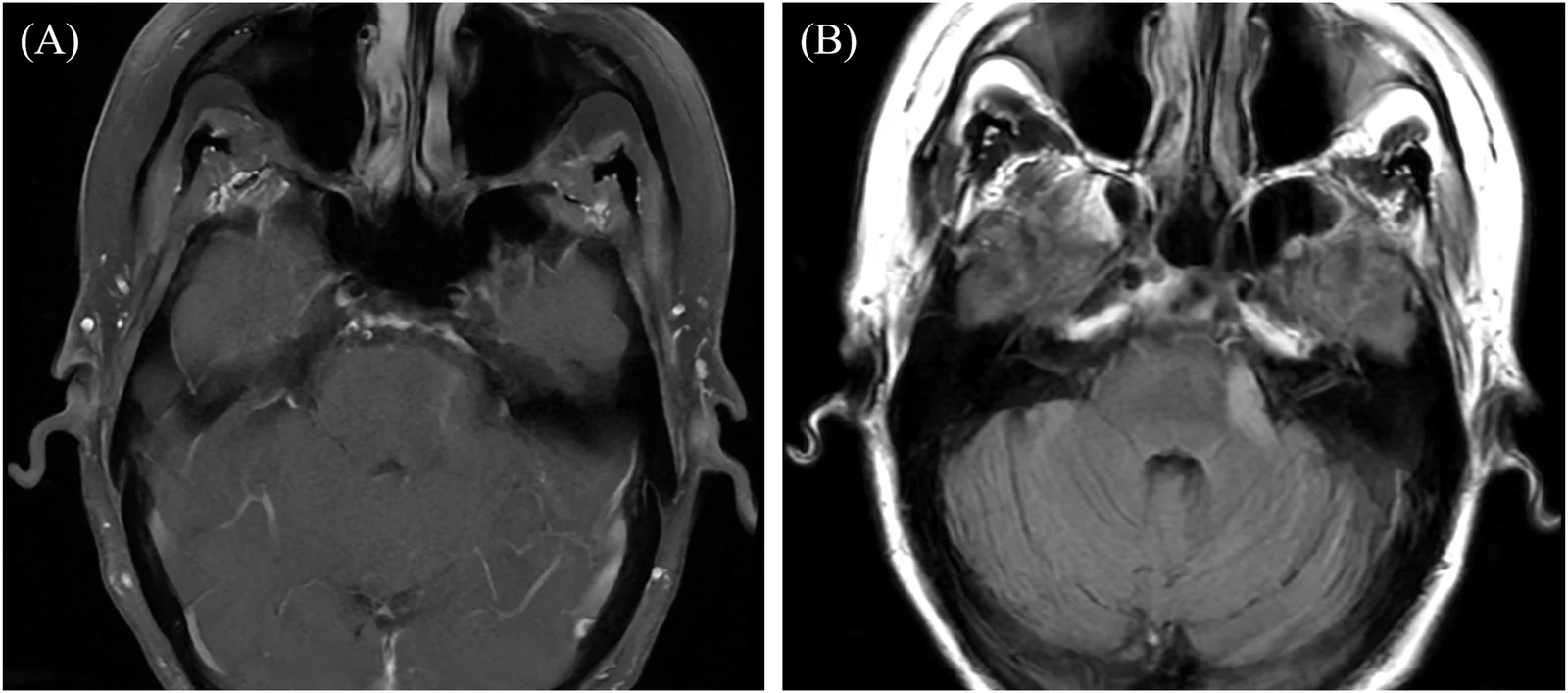
Magnetic resonance image with contrast at 10-month follow-up (A) T1-post and (B) T2- Fluid-Attenuated Inversion Recovery diminished signal in the lateral pons on the left side of the root entry zone after 3 doses of bevacicumab with no improvement in symptoms.

MRI with contrast at 6-month follow-up demonstrated lateral pontine inflammation, concerning for radiation necrosis. At 7-month follow-up, the BNI was classified as IIIa, with left facial numbness, loss of taste, dry eyes, dry mouth, and reduced hearing with tinnitus. The patient had an auditory exam with no issues. The patient increased gabapentin to 4 times daily with baclofen concurrently. Decadron 4 mg twice daily was prescribed with taper instruction. At 8-month follow-up, the BNI was classified as IV, with onset of blurry vision, left facial pressure and pain, loss of taste, dry eyes, dry mouth, and worsening tinnitus. The patient was instructed to continue on gabapentin and baclofen and to stop Decadron. MRI with contrast at 8-month follow-up yielded increased signal intensity in the lateral pons on the left side of the root entry zone (Fig. 2A). There was an interval greater area of enhancement and greater area of surrounding Fluid-Attenuated Inversion Recovery (FLAIR) hyperintensity at the pons root entry zone of the trigeminal nerve as well as a focal enhancement of the very proximal root of the left trigeminal nerve (Fig. 2B). The T1-post and T2-FLAIR signal matched with the 1200 cGy isodose line (Fig. 2C). These findings were believed to represent progression from the MRI at 6-month follow-up; thus, a diagnosis of radiation necrosis and radiation-induced neurotoxicity was made. At 9-month follow up, the patient was recommended initiation of bevacizumab (Avastin) 10 mg/kg every 2 weeks for 4 to 6 doses, with follow-up MRI after completion of 4 doses. At 10-month follow-up, the patient had completed 3 doses of bevacicumab with no improvement in symptoms. MRI with contrast at 10-month follow-up yielded diminished T1-post and T2-FLAIR signal in the lateral pons on the left side of the root entry zone (Fig. 3A and 3B).

## Discussion

In a similar case presented in the literature, Kemp et al^5^ report neurologic deficit from late onset MS, in a 65-year-old woman, after TN SRS. At 3.5 months post-TN SRS, the patient developed ataxia and left leg paraesthesiae and brain MRI showed altered signal and enhancement of the brainstem at the level of the right trigeminal root entry zone. The symptoms remitted following treatment with intravenous methylprednisolone, however, 10 months post-SRS, the patient developed gait ataxia and left lower limb weakness. MRI showed persistent T2 changes at the root entry zone and multiple new nonenhancing white matter lesions in the cerebrum and spinal cord; and oligoclonal bands were present in the cerebrospinal fluid but not serum. The report hypothesized that breakdown in the blood brain barrier secondary to SRS may have triggered a vigorous local inflammatory response. Friedman et al^12^ evaluated radiological responses for 26 patients who had been treated with doses ranging between 7000 and 9000 cGy targeting either the root entry zone (21 patients) or the retrogasserian portion of the nerve (5 patients). MRI was performed at 3 to 6 months after SRS in 21 patients. In 2 cases with MS, abnormal signal and enhancement of the brainstem and/or trigeminal nerve was seen, without any clinical complications. Previous anecdotal reports of radiation neurotoxicity in patients with MS include nerve palsies and hydrocephalus, as well as death due to progressive demyelination.^5^^,^^13^, ^14^, ^15^ Increased sensitivity to radiation-induced injury in patients with MS has been observed, possible explanations include an impaired ability to reverse radiation-induced oligodendrocyte dysfunction and demyelination, a genetic mutation associated with radiation sensitivity, and a differential effect of radiation on T-cell subtypes within demyelinating lesions.^12^^,^^16^, ^17^, ^18^

We hypothesize for our patient that an MS diagnosis coupled with a higher brainstem dose caused the irregular enhancement of the brainstem and development of neurologic deficits following TN SRS treatment. Table 1 lists the patient and dosimetric characteristics for all TN SRS patients treated at our clinic to date. The average brainstem D0.035cc for 22 TN SRS patients was 3100 ± 600 cGy, average D_max_ was 6400 ± 1200 cGy, and average volume receiving 1000 cGy was 0.34 ± 0.08 cm^3^. The patient presented in the current report had the second highest brainstem dose delivered in our clinic. The patient with the highest brainstem dose of D0.035cc at 4439.9 cGy has an MS diagnosis and at 14 months posttreatment has not experienced any neurologic deficits currently. TN SRS can result in dose to the brainstem that exceeds conventional dose constraints. High maximum doses are seen, but dose-volume histogram analysis shows rapid dose falloff with a very small volume of brainstem receiving above 1000 cGy. The result of TN radiosurgery suggests that a very small volume of the brainstem can tolerate a drastically high dose without suffering a severe clinical injury. The steep dose gradient in TN radiosurgery plays a key role in the low toxicity experienced by the brainstem.^19^, ^20^ The most common complication with TN radiosurgery is facial numbness, which is considered an endpoint of mild neuropathy associated with brainstem dose.^9^ Reporting of brainstem dose for TN SRS varies considerably across the literature with no consensus on a dose constraint and D_max_ ranging up to 7790 cGy.^19^, ^20^, ^21^, ^22^, ^23^, ^24^ A higher dose to the brainstem may result either from targeting a more proximal portion of the trigeminal nerve, from a shorter length of the nerve, or both, which results in a higher level of exposure to sensory fibers.^24^, ^25^, ^26^, ^27^ The best portion of the nerve to target remains controversial with most authors suggesting that more proximal targets induce more hypoesthesia but better pain relief,^24^, ^25^, ^26^^,^^28^, ^29^ whereas others did not find any relationship between the position of the target and outcome.^30^ A study by Ortholan et al^24^ compared Linac-based TN SRS using 5-mm or 6-mm shots, with an average brainstem D0.035cc of 1260 and 2130 cGy respectively. The study found that patients in the 6-mm group had much higher pain-free rates but also higher hypesthesia rates, especially when brainstem D0.035cc was higher than 2500 cGy. This study highlights the importance of brainstem dose optimization to achieve the best pain resolution rates while also limiting development of posttreatment hypesthesia. A large series of 329 patients treated with Linac-based TN SRS reported by Debono et al,^31^ used a 6-mm shot with a more proximal target on the plexus triangularis. The authors reported a low rate of bothersome hypesthesia (BNI grades III-IV) and hypothesized that this low rate may be related to a lower dose to the brainstem.

**Table 1:** Patient and dosimetric characteristics for all TN SRS patients treated at Jersey Shore University Medical Center. The patient presented in the current report is listed as patient 19 and had the second highest brainstem dose delivered.

Patient	Brainstem, D0.035cc	Brainstem, Dmax	Brainstem, V1000cGy	BNI before SRS	BNI after SRS	Last follow-up	Hypesthesia	MS diagnosis
	(cGy)	(cGy)	(cm^3^)			(d)		
1	1981.6	3988.68	0.206	IIIb	II	595	Numbness	
2	3308.1	6249.29	0.398	IV	V	483	Numbness	
3	2970.4	6341	0.317	IIIb	I	545	Numbness	
4	3264.3	6686.44	0.411	IIIb	II	105		
5	3787.2	7406.69	0.428	IIIb	I	677	Numbness	
6	3062.8	5814.32	0.43	IIIb	II	127		MS
7	2002.4	3409.2	0.249	IIIb	IIIb	335		
8	2638.9	6258.39	0.259	IV	II	561	Numbness	MS
9	4439.9	8084.93	0.48	IV	IIIa	420	Numbness	MS
10	3572.5	6853.51	0.351	IV	IIIb	28		
11	3231.3	5886.07	0.341	IV	IIIa	295	Numbness	MS
12	2144.3	4899.89	0.195	IIIb	II	157	Numbness	
13	3344.5	7041.21	0.361	IIIb	IIIa	206		MS
14	2867	6205.22	0.277	IV	IV	332	Numbness	
15	3232.4	6351.85	0.266	IIIb	I	119		
16	3088.8	5812.18	0.348	IV	IIIa	64		
17	3819.8	7342.94	0.373	IV	IIIb	647		
18	3106.2	7220.82	0.286	IIIb	IIIa	402		MS
19	4140.4	7874.77	0.446	IV	IV	319	Numbness	MS
20	3943.6	8086.04	0.399	IV	IIIa	120		MS
21	2589.4	7181.86	0.276	V	IV	186		
22	2866.4	5840.46	0.316	IV	IIIb	32		MS
Average:	3200	6400	0.34		Median:	307		
SD:	600	1200	0.08		IQR (p25-p75):	121.75-467.25		

*Abbreviations:* BNI = Modified Barrow Neurological Institute pain scale classification; D0.035cc = dose to 0.035 cm^3^ of brainstem; Dmax = maximum point dose to brainstem; MS = multiple sclerosis; SRS = stereotactic radiosurgery; TN = trigeminal neuralgia; V1000cGy = brainstem volume receiving 1000 cGy.

Based on our experience from the patient reported herein, we propose a brainstem dose constraint of D0.035cc < 3400 cGy for MS TN SRS patients. We have established a treatment planning policy using beam path 10 or above to keep the dose more conformal to the target and reduce brainstem dose. Although we continue to treat patients who have MS with TN SRS, we now specifically warn that there is an undefined risk of exacerbation of MS and brainstem neurotoxicity with this treatment.

## Disclosures

Timothy Chen reports a relationship with ZAP Surgical Systems, Inc that includes: consulting or advisory. Shabbar Danish reports a relationship with ZAP Surgical Systems, Inc that includes: consulting or advisory. If there are other authors, they declare that they have no known competing financial interests or personal relationships that could have appeared to influence the work reported in this paper.
